# Size‐selective mortality evolutionarily alters collective behaviour in response to predation risk in a zebrafish ( *Danio rerio* ) harvest‐induced selection model

**DOI:** 10.1111/jfb.70350

**Published:** 2026-02-10

**Authors:** Tamal Roy, Daniel João Costa Pereira de Faria, Robert Arlinghaus

**Affiliations:** ^1^ Division of Integrative Fisheries Management, Faculty of Life Sciences and Integrative Research Institute on Transformation of Human‐Environment Systems (IRI THESys) Humboldt‐Universität zu Berlin Berlin Germany; ^2^ Department of Fish Biology, Fisheries and Aquaculture Leibniz Institute of Freshwater Ecology and Inland Fisheries Berlin Germany; ^3^ Science of Intelligence Research Cluster of Excellence Berlin Germany

**Keywords:** boldness, experimental evolution, fish predation, fisheries‐induced selection, group behaviour, learning

## Abstract

Intensive multigenerational size‐selective mortality has been found to alter collective properties like shoaling via evolutionary adaptations of individual‐level behavioural traits. For example, experimental populations of zebrafish (*Danio rerio*) experiencing intensive large size‐selective mortality over multiple generations developed less cohesive shoaling in the laboratory, presumably because these fish were more attentive to environmental rather than social cues. By contrast, zebrafish exposed to small size‐selective mortality evolved increased group cohesion. However, these studies were conducted in the absence of explicit predation risk by a live predator. In this study, we examined if size‐selective mortality led to divergent responses in shoaling behaviour in the same zebrafish selection lines in the presence and absence of a live predator. The large line generated via size‐selective harvest of smaller fish over five generations formed significantly more cohesive shoals than the control line, independent of whether a live predator was present or not. By contrast, the small line generated by size‐selective mortality of large fish over five generations did not differ from controls in their shoaling behaviour in the presence or absence of a live predator. All zebrafish groups generally formed more cohesive shoals in the presence of a predator and became more dispersive over a 2‐week experimental period. We conclude that the systematic removal of smallest individuals, thereby saving the largest fish from harvest, evolutionarily fosters increased group cohesiveness. These evolutionary adaptations might affect natural predation mortality and catchability by fishing gears.

## 1 INTRODUCTION

Harvesting of wild fish populations is typically size‐selective, meaning that fish of certain size‐classes are preferentially removed by humans (Heino et al., 2015; Jørgensen et al., 2007; Kuparinen et al., 2009). Most fishing gears capture and harvest the largest size classes thereby sparing the smaller individuals, a pattern known as positive size‐selective harvest (Kuparinen et al., 2009). Alternative size‐selectivity may occur in, for example, recreational fisheries managed with a maximum‐size limit where the smaller fish are harvested, sparing the larger individuals (negative size‐selective harvest) (Pierce, 2010). Positive size‐selective harvesting has previously been shown to evolutionarily alter life histories (Heino et al., 2015) as well as a range of behavioural traits such as boldness and activity (Andersen et al., 2018; Monk et al., 2021), shoaling (Guerra et al., 2020; Sbragaglia, Jolles, et al., 2021; Sbragaglia, Klamser, et al., 2022) and learning and decision‐making (Hessenauer et al., 2016; Roy et al., 2023; Roy et al., 2024) in fish. Shoaling in social fish is an important antipredation tactic (Krause & Ruxton, 2002). Therefore, harvest‐induced evolutionary changes in shoaling behaviour can have repercussions for how fish shoals react to predators, potentially affecting natural mortality (Sbragaglia, Jolles, et al., 2021; Sbragaglia, Klamser, et al., 2022).

Predation is a key selection pressure that drives shoaling behaviour in fish (Pitcher, 1993). Fish in bigger and more cohesive groups can dilute their individual risk of predation by causing attack abatement and confusion to predators (Landeau & Terborgh, 1986; Turner & Pitcher, 1986). In support, fish from high predation habitats have been found to form bigger and tighter shoals (European minnows, *Phoxinus phoxinus* L. 1758: Orpwood et al., 2008; guppies *Poecilia reticulata* Peters 1859: Ioannou et al., 2017), and fish exposed to high predation risk are known to form more cohesive groups with stable social relationships (guppies: Heathcote et al., 2017; mosquitofish *Gambusia holbrooki* Girard 1859: Wilson et al., 2022). Fish in groups can also intensify vigilance and become shyer under increased predation risk (mosquitofish: Ward et al., 2011; juvenile perch *Perca fluviatilis* L. 1758: Goldenberg et al., 2014). Yet, what is beneficial against natural predators might turn maladaptive in fisheries because being in cohesive groups increases the risk of detection by humans through echosounders, which can increase harvest probability via trawls and seines (Croft et al., 2003; Sbragaglia, Jolles, et al., 2021).

Intensive size‐selective mortality common in capture fisheries may induce changes in shoaling behaviour through different mechanisms (Guerra et al., 2020; Sbragaglia, Jolles, et al., 2021). For example, positive size‐selective mortality may evolutionarily alter individual vigilance (Sbragaglia, Klamser, et al., 2022) by favouring individuals that are shyer, less explorative, less active and less willing to forage, and more attentive to cues from predators (Andersen et al., 2018). These behavioural changes can alter social interactions among individuals in groups, which can have impacts on collective behaviour (Couzin et al., 2002; Jolles et al., 2017). There is experimental evidence of changes in shoal cohesion in response to multigenerational size‐selective mortality in zebrafish *Danio rerio* (Hamilton 1822) experimental harvest evolution model (Sbragaglia, Klamser, et al., 2022; Sbragaglia, Roy, et al., 2022). Studies using other experimental harvest models have tested the effects of size‐selective mortality on boldness and foraging behaviour (Diaz Pauli et al., 2019; Diaz Pauli et al., 2023; Evangelista et al., 2021; Walsh et al., 2006), but not on shoaling. In experimental zebrafish populations exposed to positive size‐selective mortality over five generations, increased vigilance and decreased group cohesion were found, whereas those zebrafish exposed to negative size‐selective mortality evolved increased group cohesion (Sbragaglia, Klamser, et al., 2022; Sbragaglia, Roy, et al., 2022). However, the experiments behind these findings were conducted in the absence of explicit predation risk by a real fish predator (Sbragaglia, Klamser, et al., 2022; Sbragaglia, Roy, et al., 2022). The exact configuration of the test environment and the level of predation risk the focal fish experience through various cues (chemical and visual) affect behavioural expression in experiments (Fox et al., 2025; Klefoth et al., 2012; Roy & Arlinghaus, 2022). Live predatory fishes exert chemical and visual cues that would signal more explicit predation risk compared to, for example, a simulated aerial predator as used by Sbragaglia, Lopez‐Olmeda, et al. (2021) and Roy and Arlinghaus (2022), which only sends visual cues. Therefore, it is worth testing if the same pattern reported in past studies with the experimental zebrafish selection lines can be replicated when zebrafish groups sense predation risk by live predators.

We examined how exposure to multigenerational size‐selective mortality in zebrafish led to differences in shoal cohesion in the absence and presence of a live fish predator. To that end, three selection lines of zebrafish were used that are evolutionarily adapted to selective mortality of either the largest or the smallest fish for five generations (Uusi‐Heikkilä et al., 2015). Previous studies showed evolved differences in shoaling and collective risk‐taking behaviours among these selection lines. The large line zebrafish where the smallest individuals were harvested were found to be consistently bold by feeding on the surface after attack by a simulated aerial predator, whereas the small line zebrafish, where the largest individuals were harvested, did not differ in boldness from the control line (Roy & Arlinghaus, 2022). In the only study conducted so far in the presence of a live predatory fish, the selection lines did not differ in boldness when exposed to different predatory cues (visual, chemical or both), but there was no estimate of change in shoal cohesion along the gradient of predation (Roy & Arlinghaus, 2022). In a test of shoaling behaviour, the small line zebrafish have previously been found to form less cohesive groups, whereas the large line fish formed more cohesive groups compared to the control line in an open arena without food (Sbragaglia, Klamser, et al., 2022). However, whether these differences in shoaling behaviour are also expressed in the presence of a live predator is currently not known.

We exposed groups of eight zebrafish from the three selection lines to two conditions; a control where there was no predator present, and a treatment where there was a convict cichlid *Amantitlania nigrofasciata* (Günther 1867) in the same test tank, over a period of 2 weeks. Similar to Sbragaglia, Klamser, et al. (2022), we measured interindividual distances (IIDs) among fish groups as an estimate of grouping tendency. We hypothesized (H1) that the small line fish would form less cohesive shoals compared to the control line in both presence and absence of a live predator because these fish are more vigilant and pay more attention to environmental cues rather than social cues (Sbragaglia, Klamser, et al., 2022). We also hypothesized (H2) that the large line fish would form more cohesive shoals than the control line in both presence and absence of a live predator because these fish are less vigilant, pay more attention to social cues and respond less intensively to environmental cues (Sbragaglia, Klamser, et al., 2022).

## 2 MATERIALS AND METHODS

### 2.1 Experimental fish

Zebrafish from three selection lines (small, control and large), each with two replicates, were used in the present study (see Uusi‐Heikkilä et al., 2015 for details). The small line was generated by removing 75% of the largest individuals per generation from the parental population while allowing the remaining 25% to reproduce (Uusi‐Heikkilä et al., 2015). In a similar way, the large and control lines were generated by removing 75% of the smallest and 75% of random individuals (random for size) per generation (Uusi‐Heikkilä et al., 2015). Fish were harvested when 50% of the control line was mature (Uusi‐Heikkilä et al., 2015). This size‐selective harvesting was repeated for five consecutive generations (F_1_ to F_5_) followed by 10 generations (F_6_ to F_15_) without any further size‐selection, so that evolutionarily fixed phenotypic responses were testable. All stock populations from F_1_ to F_15_ were housed in cylindrical tanks (fibre–polyester, 320 L) in the same recirculation system at a density of approximately 1400 fish per tank in standard conditions (water temperature = 27°C, dissolved oxygen = 8.3 ± 0.3 mg/L) under 12:12 Light:Dark cycle, and were fed ad libitum with commercial flake food thrice a day. For maintenance of the lines, small groups of randomly selected fish were reared separately and the embryos produced contributed to the new generation as detailed in previous publications (Uusi‐Heikkilä et al., 2015; Uusi‐Heikkilä et al., 2016; Uusi‐Heikkilä et al., 2017). The fish evolved (Uusi‐Heikkilä et al., 2015) and maintained key evolutionary adaptations in life history and growth up to F_13_ (Sbragaglia et al., 2019). Several studies conducted on the F_13_ (Sbragaglia et al., 2019; Sbragaglia, Lopez‐Olmeda, et al., 2021) and F_16_ (Roy et al., 2023; Roy & Arlinghaus, 2022) generations showed that beyond life‐history adaptations, the selection lines had evolved specific behavioural adaptations that continued to be expressed despite cessation of size‐selection for multiple generations.

For the present experiments, we used a total of 480 11‐month‐old adult experimental fish (80 fish × 6 lines) belonging to F_16_ and conducted collective behaviour assays in the presence or absence of predation risk. To produce the experimental fish, 150 adults were randomly selected from the F_15_ stock population of each selection line, visually sexed and allowed to breed in groups of four males and two females for a day in 5 L breeding boxes that had glasswool as a complex spawning substrate. The embryos of each replicate line were pooled and randomly stocked into 3 L bare rearing boxes at a density of eight per box. In this way, a total of 60 boxes (10 boxes per line × 6 lines) were formed and held at identical conditions, each containing eight fish. The larvae were fed with commercial fish food (TetraMin Baby) four times a day. Juveniles were fed with live artemia, and the adults were fed ad libitum with commercial flake food (TetraMin Tropical) twice a day. All fish were held in standard conditions: 27°C water temperature and 12:12 light:dark cycle in the same recirculation system. A comparison of body size of the experimental fish across selection lines at around 11 months of age showed no significant differences among the lines (Supplementary Figure S1). Collective behaviour of zebrafish was tested in the absence and presence of a live convict cichlid in separate tanks. A total of 10 cichlids, each measuring approximately 12 cm, were used in the assays. These fish were held individually in 10 glass tanks (dimension 50 × 30 × 30 cm), each with a shelter made of poly vinyl chloride (PVC) and two artificial plants. All predatory fish were housed under the same standard conditions as the zebrafish and were fed with cichlid food (Tetra Cichlid Sticks) twice a day.

### 2.2 Experimental set‐up

**FIGURE 1:**
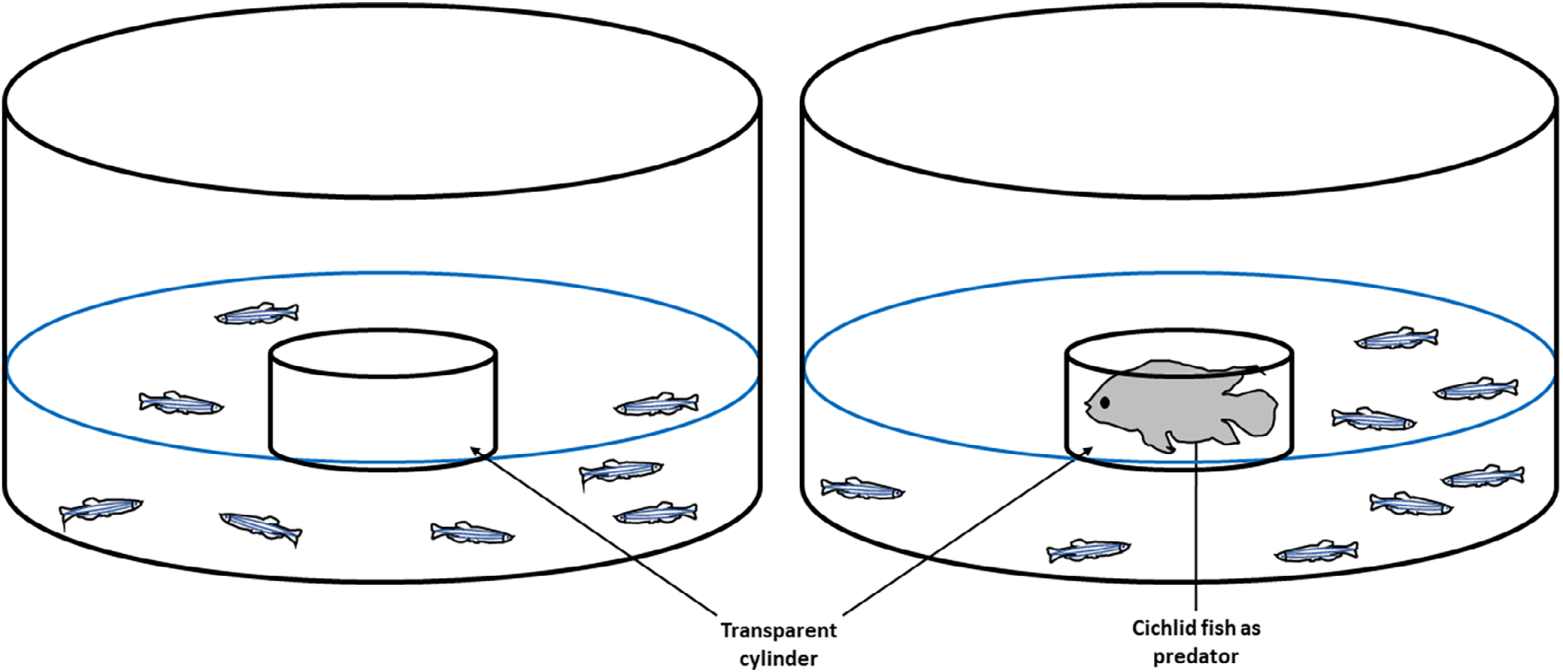
Side view of the experimental set‐up to test collective behaviour in the absence and presence of a live predator.

The experimental set‐up consisted of a circular white opaque plastic tank of 50 cm diameter that was placed on a table behind a white curtain to prevent external disturbances (Figure 1). The arena was illuminated with an overhead LED lamp, and the experiment was video recorded using a webcam (Logitech HD Pro C920; resolution: 1920 × 1080 pixels; frame rate: 30 fps) that was positioned at a distance of about 50 cm from the setup. A transparent acrylic cylinder (15 cm in diameter and 5 cm in height), open at both ends, was used to allow the exchange of both visual and chemical cues (Figure 1). The cylinder was placed in the middle of the arena where a cichlid was kept confined during the assay in the presence of a predator. In the predator‐absence assay, only the plastic cylinder was used without a predator housed in it (Figure 1).

### 2.3 Experiments

We conducted group‐level experiments across 2 weeks, running assays on four consecutive days per week. The assay in the second week assessed the repeatability of behaviour. Sixty groups were tested in two batches over four consecutive days, testing 30 groups in each batch. On days 1 and 2, half of each batch was tested in the absence and the other half in the presence of a predator. On days 3 and 4, the half that was previously tested in the absence of a predator was tested in the presence of a predator, and vice versa. The fish groups that constituted each batch were selected at random. Fish were not fed before the assays on the day of the experiment to equalize hunger levels.

Before the experiments, the tank was filled with predator‐free recirculation system water up to a level of 5 cm, and the transparent cylinder meant for confining the predator was put in the middle of the tank. The cylinder was constantly present, even when fish were tested in the absence of predator (Figure 1). When testing fish in the presence of a predator, we transferred a convict cichlid that was not fed during the previous day, into the cylinder, allowing it to acclimate for 30 min. This step was skipped when testing fish in predator absence. After this, we moved a group of eight zebrafish into a removable opaque plastic cylinder placed on one side of the arena and allowed the fish to acclimate for 5 min. Subsequently, the fish were released by removing the opaque cylinder, and then the video recording was started immediately to film the behaviour of fish for 5 min. Fish were returned to their holding after the assay and fed normally. Water in the tank was changed before testing the next group. We changed the predator after testing five zebrafish groups to avoid stressing it by prolonged confinement. Three convict cichlids were used per day for the 15 groups that were tested. Our studies were approved by the State Office for Health and Social Affairs Berlin (LaGeSo), Germany (G 0036/21), following the German Animal Welfare Law and all German bylaws regulating research on live animals. No zebrafish was exposed to direct predation.

We used IdTracker (http://www.idtracker.es) to track the position of each individual in a group during the 5 min period in the videos. From the coordinates that were generated by the programme in each frame for each fish (9000 frames, 30 frames/s), we estimated the distance between every pair of individuals in each frame. From these estimates, the average distance between each pair of fish throughout the period of the experiment was calculated, representing the mean IID.

### 2.4 Statistical analyses

Six groups; four in the small line, one in control and one in the large line, were excluded from the analysis because the software would not run the video, would return an error or the output generated had more than 1000 missing frames. To test for consistency in IID in fish groups across two repeats (weeks) and two treatment types (predator absent and present), we estimated the adjusted repeatability (Nakagawa & Schielzeth, 2010) for each selection line using the ‘rpt’ function in R. Repeatability was estimated separately for each selection line and each treatment type over the two repeat intervals. The square root–transformed measurement variable (IID) was used as the dependent variable, with Repeat (week 1 and 2) as a fixed effect and the Group ID as a random effect. 95% confidence intervals with a significance level of 5% were used as estimates of uncertainty. To test if IID differed in the absence and presence of a live predator and across time (repeats), we fitted a linear mixed effects models (lmer) using the transformed IID measure as a dependent variable, a three‐way interaction of Selection line (small, control and large), Treatment (with or without predator) and Repeat (week 1 and week 2) as fixed effects, and Group ID nested within selection line replicate as the random effect. If the interaction terms were not significant, they were removed and the model was rerun with just the main effects. Cohen's D (Cohen, 2013) was computed to quantify the effect size of selection line on IID.

All analyses were done in R version 4.4.0 (R Core Team, 2022) using ‘lmerTest’ package for constructing linear mixed effects models (Kuznetsova et al., 2017), ‘rptR’ package to calculate repeatability (Stoffel et al., 2017), ‘lsr’ package to calculate Cohen's D (Navarro, 2015) and ‘ggplot2’ package for visualization (Wickham, 2011).

## 3 RESULTS

**FIGURE 2:**
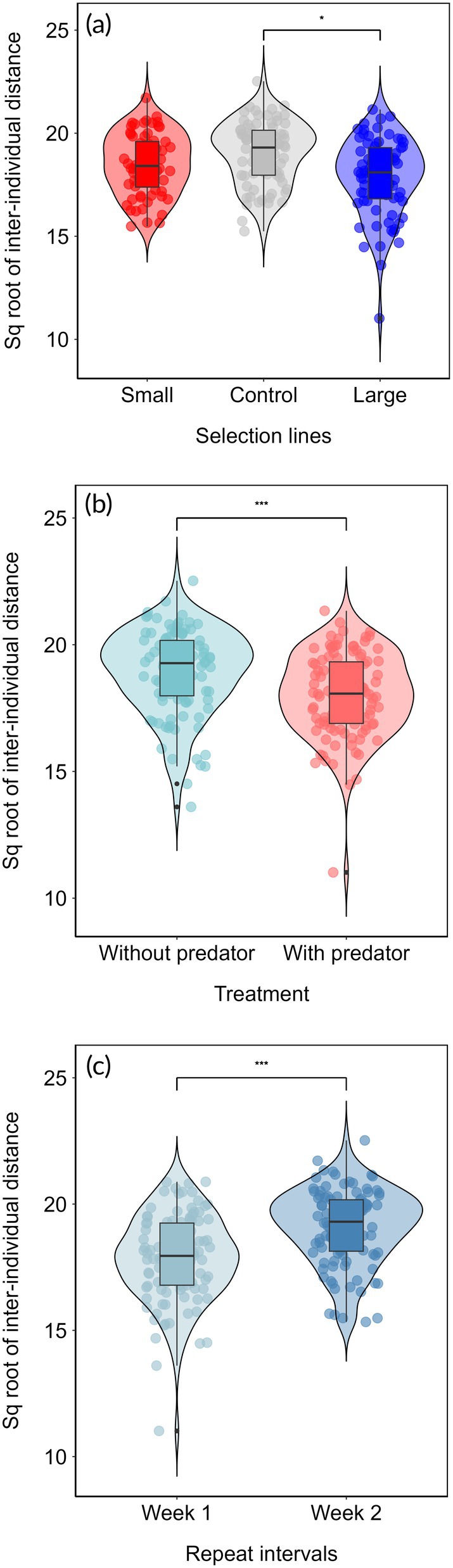
Square root of interindividual distance among fish groups (a) was significantly lower in the large line compared to the control line, (b) was significantly lower in the presence than in the absence of a predator and (c) was significantly higher during week 2 than during week 1.

The IID was significantly repeatable among the three selection lines across treatments (Table 1). This consistency indicated the presence of a collective personality; that is, groups of zebrafish were consistently more or less cohesive. In a full model considering the interactions of treatment, repeat and selection line (fixed effects), we found that IID in fish groups of the large line, but not the small line, differed significantly from the control line (large line: *t* = −2.84, *p* = 0.03; small line: *t* = −1.32, *p* = 0.23), and IID differed significantly between treatments (with/without predator; *t* = −2.88, *p* < 0.01) and repeats (week 1/2; *t* = 3.04, *p* < 0.01). Yet, there was no significant three‐way interaction of treatment, repeat and selection line on IID (Table 2a), indicating that the selection line differences were consistent across treatment and repeats rather than a function of these factors. After removing the non‐significant interaction term and rerunning the model, we found that the IID in fish groups of the large line continued to be significantly lower than the IID of the control line (*t* = −2.57, *p* = 0.01), which indicated that the large line fish formed significantly more cohesive groups with medium effect size (Cohen's *D* = 0.69) compared to controls, independent of whether or not a predator was present (Figure 2a, Table 2b). The IID among individuals in fish groups was significantly and generally lower in the presence rather than in the absence of a caged cichlid predator (*t* = −5.97, *p* < 0.001), independent of the selection line, which indicated that fish groups of all selection lines were more cohesive in the presence of a live predator compared to when the predator was absent in the test tanks (Figure 2b; Table 2b). The IID among individuals in groups also significantly increased in the second week compared to the first week (*t* = 8.40, *p* < 0.001), revealing that fish groups became less cohesive with experimental time (Figure 2c; Table 2b).

**TABLE 1:** Between‐ and within‐group variances and repeatability of interindividual distances (IIDs) in fish groups among selection lines in the absence and presence of a predator.

Selection line	*R*	Std. errors	CI	*p*
Without predator				
Small	0.39	0.20	0, 0.72	**0.05**
Control	0.34	0.20	0, 0.69	0.06^+^
Large	0.41	0.19	0, 0.72	**0.03**
With predator				
Small	0.45	0.20	0, 0.76	**0.02**
Control	0.61	0.16	0.24, 0.83	**<0.01**
Large	0.60	0.15	0.24, 0.83	**<0.01**

*Note*: Repeatability (*R*) was estimated at 95% confidence interval and 5% level of significance. Significant results are shown in bold, and marginal results are market with ‘+’.

**TABLE 2:** Results of linear mixed effects model built to compare interindividual distance among selection lines between predator absent and present treatments and over the period of 2 weeks.

Predictors	Estimates	Std. error	df	CI	*t*	*p*
(a)						
(Intercept)	19.06	0.39	6.20	18.29–19.84	48.70	**<0.001**
Line [large]	−1.57	0.55	6.20	−2.66 – −0.48	−2.84	**0.03**
Line [small]	−0.75	0.57	6.70	−1.87 – 0.37	−1.32	0.23
Treatment [with predator]	−1.04	0.36	156	−1.76 – −0.33	−2.88	**<0.01**
Repeat [Week 2]	1.10	0.36	156	0.39–1.81	3.04	**<0.01**
Line [large] × treatment [with predator]	0.33	0.51	156	−0.68 – 1.34	0.65	0.51
Line [small] × treatment [with predator]	0.08	0.53	156	−0.96 – 1.12	0.15	0.88
Line [large] × repeat [Week 2]	0.49	0.51	156	−0.52 – 1.50	0.96	0.34
Line [small] × repeat [Week 2]	−0.06	0.53	156	−1.10 – 0.97	−0.12	0.90
Treatment [with predator] × repeat [Week 2]	−0.23	0.51	156	−1.24 – 0.78	−0.45	0.65
(Line [large] × treatment [with predator]) × repeat [Week 2]	−0.05	0.72	156	−1.47 – 1.38	−0.06	0.95
(Line [small] × treatment [with predator]) × repeat [Week 2]	0.86	0.74	156	−0.61 – 2.32	1.15	0.25

(b)						
(Intercept)	18.85	0.34	3.53	18.18–19.52	55.51	**<0.001**
Line [large]	−1.17	0.46	2.88	−2.07 – −0.27	−2.57	**0.01**
Line [small]	−0.53	0.47	3.07	−1.44 – 0.39	−1.13	0.26
Treatment [with predator]	−0.89	0.15	163	−1.19 – −0.60	−5.97	**<0.001**
Repeat [Week 2]	1.26	0.15	163	0.96–1.55	8.40	**<0.001**

*Note*: (a) shows results considering interactions of fixed effects, and (b) shows results of the simplified model after removing interactions. Significant results are shown in bold.

## 4 DISCUSSION

We studied the evolutionary impact of size‐selective mortality on the plasticity of collective behaviour in zebrafish in the absence and presence of a live fish predator across two 7‐day time intervals. The shoaling behaviour in zebrafish groups was significantly repeatable across contexts, providing evidence for the presence of a collective personality. Moreover, fish groups, irrespective of their selection line identity, became more cohesive when a live predator was present. This finding is consistent with a large body of literature that has shown social fish to increase shoaling tendency and group cohesiveness in the presence of predators (Landeau & Terborgh, 1986; Pitcher, 1993; Turner & Pitcher, 1986). In support of the second hypothesis (H2), zebrafish exposed to negative size‐selection (large line) formed more cohesive shoals compared to controls. However, our results disagreed with the first hypothesis (H1), and also with findings from studies conducted without predation risk from a live predator which revealed that zebrafish exposed to positive size‐selection (small line) formed more cohesive groups (Sbragaglia, Klamser, et al., 2022; Sbragaglia, Roy, et al., 2022). Instead, the present work showed that size‐selected lines formed more cohesive shoals when a live predator was present in the test environment. Thus, the configuration of the test environment seems to modulate collective behavioural expression for the small line, whereas our findings for the large line agreed with past work in different experimental set‐ups (Sbragaglia, Klamser, et al., 2022). That the large and control line fish differed in shoal cohesion 11 generations after selection was stopped can be interpreted as an evolutionarily fixed response to five generations of size‐selective harvest.

Shoaling behaviour emerged as a collective personality trait in fish groups across all selection lines of zebrafish. This is in line with previous studies in other schooling fish species like guppies and three‐spined sticklebacks *Gasterosteus aculeatus* L. 1758 (Clark et al., 2025; Georgopoulou et al., 2022; Kim et al., 2022). The results also mirror findings of previous studies using the same selection lines of zebrafish, where collective boldness (Roy & Arlinghaus, 2022; Sbragaglia, Lopez‐Olmeda, et al., 2021) and shoal cohesiveness (Sbragaglia, Klamser, et al., 2022; Sbragaglia, Roy, et al., 2022) were found to be collective personality traits. These findings show that group‐level behaviours can be interpreted as collective personality traits in zebrafish like other social fishes.

The zebrafish line selected for large body size through negative size‐selective harvest (large line) showed increased shoal cohesion compared to the controls independent of whether a live predator was present or not in the test tank. This finding is consistent with a previous study using the same selection lines that showed that the large line fish form more cohesive shoals (Sbragaglia, Klamser, et al., 2022). In that study, the difference in IID between large and control lines in the absence of a predator had a very large effect size of 1.15 (Sbragaglia, Klamser, et al., 2022), whereas we found a medium effect size of 0.69. Different sample sizes in the two studies, variation in experimental set‐ups, presence or absence of a live predator and the use of fish from different generations (F_13_ in the previous study versus F_16_ in the current study) could be the reasons behind the different effect sizes of the two studies. The large line zebrafish formed more cohesive groups presumably because these fish are less vigilant of the environment and pay more attention to social cues in groups (Sbragaglia, Klamser, et al., 2022). Fish that pay more attention to social cues will not lose connections with conspecifics easily when they are on the move and this would lead to increased group cohesiveness (Calovi et al., 2018; Harpaz et al., 2017).

Increased shoaling, as observed in the large line fish, may have implications for food acquisition and predator avoidance in the wild. Shoaling in social fish helps in locating food (Krause & Ruxton, 2002), and we speculate that shoaling tendency might have been co‐selected with size‐at‐age that was the focal trait on which selection acted in the original harvest‐selection experiment (Uusi‐Heikkilä et al., 2015). The large line fish have consistently shown higher collective foraging propensity in previous studies (Roy & Arlinghaus, 2022; Sbragaglia, Lopez‐Olmeda, et al., 2021), and we propose that these behaviours are positively correlated with cohesive shoaling. Under natural conditions, higher shoaling tendency is also beneficial to reduce mortality from natural predators (Krause & Ruxton, 2002) through processes like attack abatement and predator confusion (Landeau & Terborgh, 1986; Pitcher, 1993; Turner & Pitcher, 1986). Therefore, the large line might suffer from lower natural mortality (Sbragaglia, Klamser, et al., 2022). Further studies that use freely swimming live predators are necessary to confirm this speculation. Increased shoaling may, however, be maladaptive in the wild under fishing conditions. Small pelagic and other social species are often targeted by trawls and seines (i.e., active gear types: Diaz Pauli & Sih, 2017) where the detection through echosounders and the subsequent capture process is aided by the schooling tendency of fish (Croft et al., 2003; Hollins et al., 2019; Thambithurai et al., 2018). Increased shoaling may, thus, result in increased capture probability of fish (Croft et al., 2003; Guerra et al., 2020; Sbragaglia, Klamser, et al., 2022).

The zebrafish line selected for small body size, as is typical in most fisheries, did not show any difference in shoal cohesion compared to the control line fish, irrespective of whether or not a live predator was present. This result contradicted a previous study that showed the small line zebrafish formed less cohesive shoals compared to the control line (Sbragaglia, Klamser, et al., 2022). As mentioned before, difference in sample sizes, test environments, predator cues and fish generations used in the study of Sbragaglia, Klamser, et al. (2022) and the present work might be the reasons behind the differences in study outcomes. However, our observation of asymmetric changes in shoaling behaviour in the large and small lines matches with that of a previous study that tested the ontogenetic change in collective boldness among the zebrafish selection lines, which revealed that the large line, and not the small line, was consistently bolder compared to the control line fish (Roy & Arlinghaus, 2022). Asymmetric response to size‐selection with only one of the size‐selected lines demonstrating the predicted changes in traits has been previously observed in other harvest evolution models with live fish (Bartuseviciute et al., 2022; Diaz Pauli et al., 2019; Le Rouzic et al., 2020; Renneville et al., 2020). The asymmetric change in shoaling behaviour of zebrafish selection lines observed in our study compared to what was observed previously by Sbragaglia, Klamser, et al. (2022) could also have been caused by partial trait recovery (Salinas et al., 2012; van Dijk et al., 2024). The study by Sbragaglia, Klamser, et al. (2022) was conducted on F_13_, whereas our study was conducted on F_16_. Three generations could be enough for shoaling behaviour of the small line fish to bounce back to levels similar to that of the control line fish groups. Indeed, in contrast to earlier work on the zebrafish lines (Roy et al., 2021; Sbragaglia et al., 2019; Uusi‐Heikkilä et al., 2015), the two size‐selected lines we used no longer differed in average body length, suggesting a recovery of growth rate. However, the large line maintained strong differences in group behaviour relative to controls despite possible trait recovery, and therefore we can rule out that systematic length differences among the fish in the two selection lines were responsible for the different study findings.

The large and small lines did not differ in shoaling behaviour based on whether a predator was present or not. Although no previous study measured group cohesiveness among the zebrafish selection lines in the presence of a live predator, Roy and Arlinghaus (2022) tested collective risk‐taking to forage (as a measure of boldness) when exposed to different cues (visual, chemical or both) from a caged convict cichlid, in F_16_ fish of the same selection lines. That study did not find differences in boldness in the three predation contexts among the selection lines, consistent with our findings that predation context does not moderate group responses among selection lines. It could be that when a live predator is present, differences in behavioural tendencies among the lines might disappear because of the mortality consequences that may follow. This argument is supported by studies in guppies where bolder individuals were more dispersive compared to shyer ones in the absence of predators (Cote et al., 2010), but these differences faded out in the presence of live predators (Cote et al., 2013).

Fish shoals generally became more cohesive in the presence of a live predator compared to when there was no predator. This is supported by previous studies on other species like the guppy, three‐spined sticklebacks and mosquitofish that showed that fish increased shoaling with increased perception of predation risk (Kozak & Boughman, 2012; Li et al., 2022; Wilson et al., 2022). Increased grouping tendency is an adaptive response to predation risk in social fishes because this helps reduce mortality risk (Krause & Ruxton, 2002; Monk et al., 2023; Pitcher et al., 1998). Our zebrafish selection lines, despite being reared in laboratory conditions, continue to be able to sense predation risk and plastically change behaviour accordingly, similar to how wild‐caught zebrafish adapt their behaviours when faced with live predators (Roy et al., 2017; Roy & Bhat, 2018a, 2018b).

Shoal cohesion decreased during the second week of assay compared to the first week, indicating that fish groups became more dispersive over experimental time. This could be because the fish groups got habituated or used to the experimental set‐up that was initially novel, but we lack a proper time control for substantiating this speculation. Previous studies, however, have shown a similar effect while measuring collective behavioural properties across different time scales. For example, in three‐spined sticklebacks, fish groups became less social and showed reduced shoal cohesion over different time scales because of acclimatization to the experimental set‐up over time (MacGregor & Ioannou, 2021). As another example in zebrafish, groups shifted from tighter schooling configuration to loose shoaling configuration due to habituation to the environment when tested repeatedly (Miller & Gerlai, 2012). A previous study that tested change in shoal cohesion across ontogenetic time points in our selection lines also found that fish shoals of the large and control selection lines became less cohesive over time (Sbragaglia, Roy, et al., 2022). However, shoaling was tested in a predator‐free environment in that study. We find that irrespective of whether a predator is present or not and independent of selection line, shoal cohesion decreased with experimental time in our zebrafish.

Our study suggests that size‐selection did not result in differences in how zebrafish groups respond to the presence or absence of predators. Even if these differences existed at some point, they were no longer detectable 11 generations after size‐selection stopped. When threats are severe, zebrafish generally behaved more cautiously and schooled tightly together to seek the safety of crowd. That said, we found the large line fish groups to be generally more cohesive, and this tendency might not only decrease natural mortality but also increase food localization and foraging in social fish (Pitcher, 1993; Pitcher et al., 1998). By contrast, the small line did not differ from controls in shoaling. That size‐selection leads to asymmetric evolutionary outcomes is a finding reported previously in harvest selection experiments in other fish species (Bartuseviciute et al., 2022; Renneville et al., 2020). If the collective behaviours we found are also expressed in wild fish populations adapted to heavy fishing pressure over multiple generations, then in fisheries where large individuals are saved from harvest, social fish may evolutionarily develop increased shoaling. Such evolutionary adaptation could reduce natural mortality (Sbragaglia, Klamser, et al., 2022) but may increase catchability to certain gear types (e.g., trawls or seines: Diaz Pauli & Sih, 2017). Investigating these hypotheses with free‐ranging predators and simulated fishing gears is of worth in the future.

## AUTHOR CONTRIBUTIONS

Tamal Roy, Daniel João Costa Pereira de Faria and Robert Arlinghaus conceived this study. Daniel João Costa Pereira de Faria conducted the experiments. Tamal Roy and Daniel João Costa Pereira de Faria analysed the data. Tamal Roy and Robert Arlinghaus wrote the manuscript with inputs from Daniel João Costa Pereira de Faria.

## FUNDING INFORMATION

Tamal Roy was supported by a postdoctoral research fellowship from Alexander von Humboldt foundation (Germany). Tamal Roy and Robert Arlinghaus were funded by the German Research Foundation under Germany's Excellence Strategy – EXC 2002/1 ‘Science of Intelligence’ – project number 390523135.

## Supporting information


**FIGURE S1.** Comparison of body size of the experimental fish (*N* = 480, 80 fish per replicate line) showed that the small and large line fish did not differ significantly from the control line in size (small line: *t* = −0.93, *p* = 0.42, large line: *t* = −1.71, *p* = 0.19).
