# Supplementary material for: Size‐selective mortality evolutionarily alters collective behaviour in response to predation risk in a zebrafish ( Danio rerio ) harvest‐induced selection model
Source: J Fish Biol. 2026 Feb 10;108(6):1865–74. doi: 10.1111/jfb.70350 (PMC13357273; doi:10.1111/jfb.70350)
Supplement: Supplementary file 1 — FIGURE S1. Comparison of body size of the experimental fish (N = 480, 80 fish per replicate line) showed that the small and large line fish did not differ significantly from the control line in size (small line: t = −0.93, p = 0.42, large line: t = −1.71, p = 0.19). [file JFB-108-1865-s001.docx]

**Supplementary Information**

**Size-selective mortality alters collective behaviour in the face of predation risk in a zebrafish (*Danio rerio*) harvest evolution model**

Tamal Roy^1,2,3^*, Daniel João Costa Pereira de Faria^1,2^ and Robert Arlinghaus^1,2,3^

^1^Division of Integrative Fisheries Management, Faculty of Life Sciences and Integrative Research Institute on Transformation of Human-Environment Systems (IRI THESys), Humboldt-Universität zu Berlin, Unter den Linden 6, 10099 Berlin, Germany

^2^Department of Fish Biology, Fisheries and Aquaculture, Leibniz Institute of Freshwater Ecology and Inland Fisheries, Müggelseedamm 310, 12587 Berlin, Germany

^3^Science of Intelligence, Research Cluster of Excellence, Marchstrasse 23, 10587 Berlin


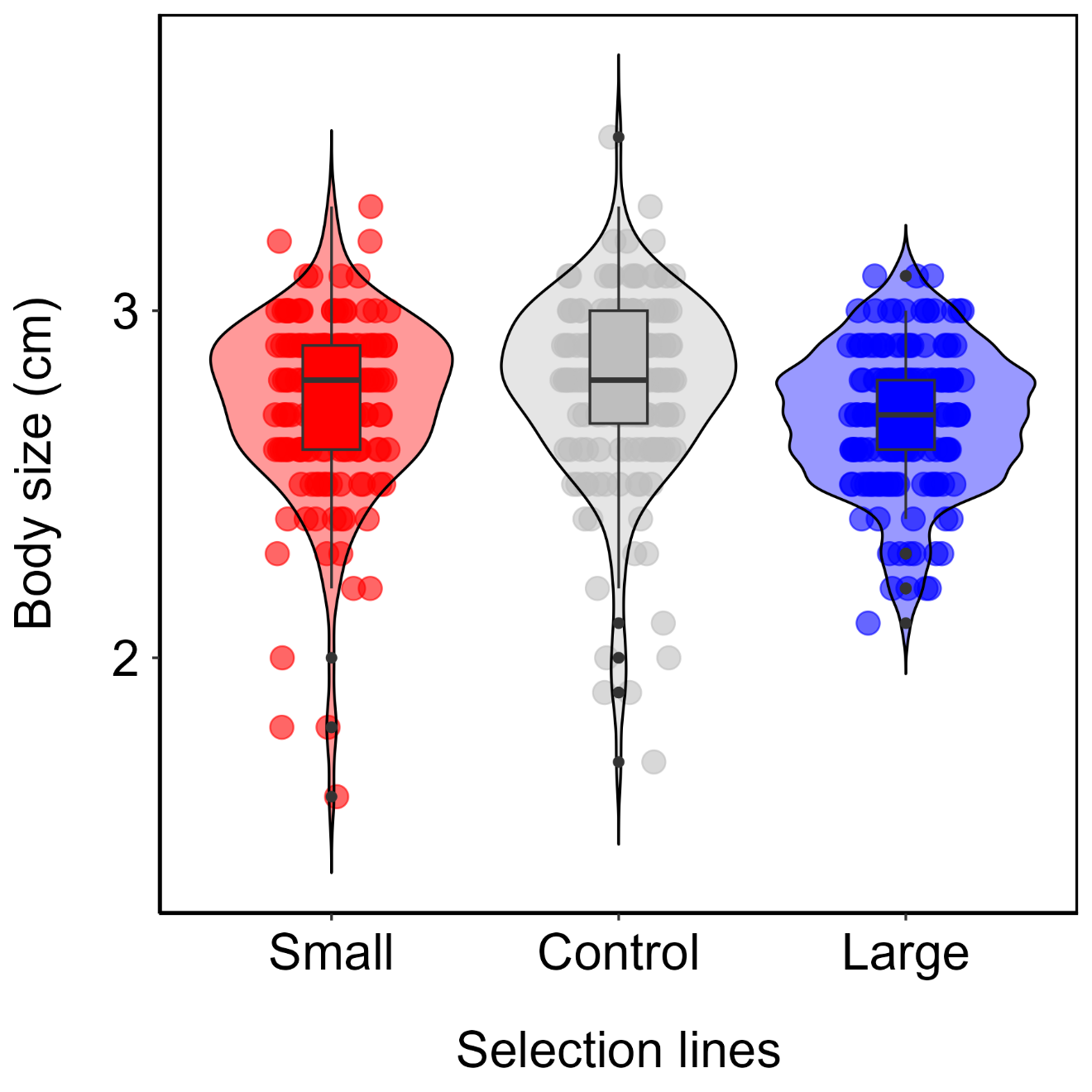


Supplementary Figure 1: Comparison of body-size of the experimental fish (N=480, 80 fish per replicate line) showed that the small and large line fish did not differ significantly from the control line in size (Small line: t=-0.93, p=0.42, Large line: t=-1.71, p=0.19).
